# Pregnancy‐specific glycoprotein 9 acts as both a transcriptional target and a regulator of the canonical TGF‐β/Smad signaling to drive breast cancer progression

**DOI:** 10.1002/ctm2.245

**Published:** 2020-12-12

**Authors:** Ying‐Ying Liu, Sa Zhang, Tian‐Jian Yu, Fang‐Lin Zhang, Fan Yang, Yan‐Ni Huang, Ding Ma, Guang‐Yu Liu, Zhi‐Ming Shao, Da‐Qiang Li

**Affiliations:** ^1^ Fudan University Shanghai Cancer Center and Shanghai Key Laboratory of Medical Epigenetics International Co‐laboratory of Medical Epigenetics and Metabolism Ministry of Science and Technology Institutes of Biomedical Sciences Fudan University Shanghai China; ^2^ Cancer Institute Shanghai Medical College, Fudan University Shanghai China; ^3^ Department of Oncology Shanghai Medical College, Fudan University Shanghai China; ^4^ Department of Breast Surgery Shanghai Medical College, Fudan University Shanghai China; ^5^ Shanghai Key Laboratory of Breast Cancer Shanghai Medical College, Fudan University Shanghai China

**Keywords:** breast cancer, epithelial‐mesenchymal transition, pregnancy‐specific glycoprotein, Smad, TGF‐β signaling

## Abstract

Pregnancy‐specific glycoprotein 9 (PSG9) is a placental glycoprotein essential for the maintenance of normal gestation in mammals. Bioinformatics analysis of multiple publicly available datasets revealed aberrant PSG9 expression in breast tumors, but its functional and mechanistic role in breast cancer remains unexplored. Here, we report that PSG9 expression levels were elevated in tumor tissues and plasma specimens from breast cancer patients, and were associated with poor prognosis. Gain‐ or loss‐of‐function studies demonstrated that PSG9 promoted breast cancer cell proliferation, migration, and invasion*in vitro*, and enhanced tumor growth and lung colonization *in vivo*. Mechanistically, transforming growth factor‐β1 (TGF‐β1) transcriptionally activated PSG9 expression through enhancing the enrichment of Smad3 and Smad4 onto *PSG9* promoter regions containing two putative Smad‐binding elements (SBEs). Mutation of both SBEs in the *PSG9* promoter, or knockdown of TGF‐β receptor 1 (TGFBR1), TGFBR2, Smad3, or Smad4 impaired the ability of TGF‐β1 to induce PSG9 expression. Consequently, PSG9 contributed to TGF‐β1‐induced epithelial‐mesenchymal transition (EMT) and breast cancer cell migration and invasion. Moreover, PSG9 enhanced the stability of Smad2, Smad3, and Smad4 proteins by blocking their proteasomal degradation, and regulated the expression of TGF‐β1 target genes involved in EMT and breast cancer progression, thus further amplifying the canonical TGF‐β/Smad signaling in breast cancer cells. Collectively, these findings establish PSG9 as a novel player in breast cancer progression*via* hijacking the canonical TGF‐β/Smad signaling, and identify PSG9 as a potential plasma biomarker for the early detection of breast cancer.

## Graphical abstract

Our findings establish PSG9 as a novel player in breast cancer progression *via* hijacking the canonical TGF‐β/Smad signaling and identify PSG9 as a potential plasma biomarker for the early detection of breast cancer.

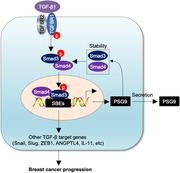

**Abbreviations**
ANGPTL4: angiopoietin‐like 4
EMT: epithelial‐mesenchymal transition
IL‐11: interleukin 11
PSG9: pregnancy‐specific glycoprotein 9
SBE: Smad‐binding element
TGFBR: TGF‐β receptor
TGF‐β: transforming growth factor‐β

## 1 INTRODUCTION

Breast cancer is the most commonly diagnosed cancer and the second leading cause of cancer‐related death among females worldwide.^1^ Despite considerable progress in the management of localized lesions, metastatic breast cancer remains incurable and contributes to the overwhelming majority of breast cancer‐related deaths.^2^ Accumulating evidence indicates that aberrant activation of oncogenic epithelial‐mesenchymal transition (EMT) program is closely associated with the acquisition of the invasive and metastatic phenotype of breast cancer cells.^3^ The EMT event is initiated and controlled by signaling pathways that respond to a variety of extracellular and intracellular signals. Among these, transforming growth factor‐β (TGF‐β) is a potent inducer of EMT in breast cancer cells through activating both canonical and noncanonical pathways.^4^, ^5^ In the canonical pathway, TGF‐β binds to and activates a transmembrane heterodimeric complex consisting of two serine/threonine kinase receptors (TGF‐β receptor 1 [TGFBR1] and TGFBR2), resulting in phosphorylation of intracellular effectors Smad2 and Smad3. Phosphorylated Smad2 and Smad3 form a complex with the common mediator Smad4 and then translocate into the nucleus, where they selectively regulate TGF‐β target gene expression through binding to the consensus Smad‐binding element (SBE) in their promoters.^6^, ^7^ In addition, TGF‐β also modulates EMT network through activating noncanonical pathways, including extracellular regulated kinase (ERK), c‐Jun N‐terminal kinase (JNK), and phosphoinositide 3‐kinase (PI3K).^8^ Consistent with its role in EMT, an upregulation of TGF‐β signaling is closely associated with breast cancer progression and poor prognosis.^9^ Consequently, blockade of TGF‐β signaling suppresses EMT and breast cancer cell motility, invasion, and metastasis.^10^, ^11^, ^12^, ^13^ Despite the functional and clinical importance of TGF‐β network in breast cancer progression, its mechanism of action still remains unclear.

The pregnancy‐specific glycoproteins (PSGs) are the most abundant placental proteins in the maternal bloodstream during pregnancy in rodents and primates.^14^ Human PSG proteins, encoded by 11 highly conserved PSG genes (PSG1‐PSG11), are secreted by placental syncytiotrophoblast and are implicated in immunoregulation, thromboregulation, and angiogenesis during pregnancy.^14^, ^15^, ^16^, ^17^ The PSG plasma levels are detectable around day 14 postfertilization and reach the maximum at the third trimester of pregnancy.^18^ Low PSG levels in maternal circulation are associated with adverse pregnancy outcomes, including fetal growth restriction, preterm delivery, and pre‐eclampsia,^19^, ^20^, ^21^, ^22^ underscoring the importance of PSGs in the establishment of a successful pregnancy. Accumulating evidence shows that multiple human PSG proteins can induce secretion and activation of TGF‐β at the maternal‐fetal interface to establish an appropriate immunosuppressive and inflammatory environment during pregnancy.^14^, ^16^, ^23^, ^24^, ^25^, ^26^ However, whether TGF‐β1 in turn regulates PSGs and PSGs modulate the core components of the TGFBR/Smad pathway remain unknown.

Analyses of multiple publicly available databases^27^ indicate that pregnancy‐specific glycoprotein 9 (PSG9), but not other PSGs, is significantly upregulated in breast tumors, but its functional and mechanistic role in breast cancer remains unexplored. In this study, we provide the first evidence that PSG9 expression levels are associated with the metastatic phenotype of breast tumors and shorter patient survival. Functional and mechanistic investigations demonstrated that PSG9 promotes breast cancer progression by acting as both a direct transcriptional target and a positive regulator of the canonical TGF‐β/Smad signaling.

## 2 MATERIALS AND METHODS

### 2.1 Cell cultures and treatments

Human breast cancer cell lines (MCF‐7, HCC1806, HCC1937, SUM159, Hs578T, MDA‐MB‐453, and BT549), normal human mammary epithelial cell lines (HMEC and HBL100), and human embryonic kidney 293T cell line (HEK293T) were obtained from the Cell Bank of the Chinese Academy of Sciences (Shanghai, China). MDA‐MB‐231 (MDA‐231)‐derived LM2‐4173 and LM2‐4175 cells were kindly provided by Guohong Hu (University of Chinese Academy of Sciences, Shanghai, China). Both cell lines have enhanced lung metastasis potential compared to their parental counterpart.^28^ The isogenic MCF10 cell series, including immortalized normal breast epithelial MCF10A, premalignant MCF10AT, ductal carcinoma in situ MCF10DCIS, and metastatic MCF10CA1d cell lines,^29^, ^30^, ^31^, ^32^ were originally obtained from the Cell Lines Resource of Karmanos Cancer Institute (Detroit, MI) and maintained at Shanghai Key Laboratory of Breast Cancer. The 67NR, 168FARN, 4T07, and 4T1 cell lines were initially derived from a spontaneous breast tumor growing in a BALB/c mouse.^33^ Those four isogenic murine breast cancer cell lines were originally provided by Dr Fred Miller (Karmanos Cancer Institute) and maintained at Shanghai Key Laboratory of Breast Cancer. All cell lines were routinely authenticated by detection of mycoplasma, cell vitality, and short tandem repeat (STR) profiling. Cell lines were expanded and frozen immediately into numerous aliquots after arrival. The cells revived from the frozen stock were used within 10‐15 passages and a period of 6 months.

MCF10A sublines were cultured as described in detail previously.^29^, ^30^, ^31^, ^32^ Other cell lines were cultured in DMEM medium (BasalMedia, #L110) supplemented with 10% fetal bovine serum (ExCell Biol, #FSS500) and 1% penicillin‐streptomycin (BasalMedia, #S110B). For TGF‐β1 treatment, cells were serum‐starved for 24 hours and then incubated with or without 10 ng/mL recombinant human TGF‐β1 (Cell Signaling Technology, #8915LF) for the indicated times. Unless otherwise stated, all reagents were purchased from Sigma‐Aldrich.

### 2.2 Expression vectors, plasmid transfection, and lentiviral infection

cDNAs for PSG9 (#CH879913), Smad2 (#CH809987), Smad3 (#CH881773), and Smad4 (#CH889836) in pEnter vector were obtained from Vigene Biosciences, and then subcloned into the lentiviral vector pCDH‐CMV‐MCS‐EF1‐Puro (System Biosciences, #CD510B‐1) to generate HA‐PSG9, Flag‐Smad2, Flag‐Smad3, or Flag‐Smad4, respectively, using CloneEZ PCR Cloning Kit (Genscript, #L00339). Expression vector encoding V5‐ubiquitin has been described previously.^34^ Short hairpin RNAs (shRNAs) targeting PSG9 (shPSG9) in pGIPZ lentiviral vector and corresponding negative control (shNC) were purchased from Dharmacon (#RHS4430). The *PSG9* promoter region was amplified by PCR and cloned into a pGL3‐basic luciferase reporter vector (Promega, #E1751). Mutations of the SBEs in the *PSG9* promoter were performed by PCR‐based methods. All construct sequences were verified by DNA sequencing. The primers used for molecular cloning are provided in Table S1. Transient plasmid transfection was performed using Neofect DNA transfection reagent (TengyiBio, #TF201201) according to the manufacturer's protocol. Lentiviral infection and generation of stable cell lines were carried out as described previously.^34^, ^35^

### 2.3 Small interfering RNAs (siRNAs) and transfection

Specific siRNAs targeting TGFBR1, TGFBR2, Smad2, Smad3, Smad4, and corresponding negative control siRNAs (siNC) were purchased from GenePharma (Shanghai, China), and their targeting sequences were listed in Table S2. The siRNA duplexes were transfected into cells using Lipofectamine 2000 transfection reagents (Invitrogen, #11668019) following the manufacturer's instructions. Knockdown efficiency was determined by immunoblotting analysis after 48 hours of transfection.

### 2.4 Antibodies, immunoblotting, and immunoprecipitation assays

All primary antibodies used in this study are listed in Table S3. The secondary antibodies for immunoblotting assays and immunofluorescent staining were purchased from Cell Signaling Technology. Immunoblotting analysis and immunoprecipitation assays were performed following standard protocols as described previously in detail.^34^, ^35^ The optical density of immunoblotting bands was quantified using ImageJ program (http://rsb.info.nih.gov/ij/), and was normalized to the internal control vinculin.

### 2.5 qPCR and ChIP‐qPCR

Total RNA was isolated using TRIzol reagent (Invitrogen, #15596018), and 1 μg of RNA was subjected to cDNA synthesis using PrimeScript RT Master Mix (Takara, #RR036). Quantitative real‐time PCR (qPCR) was performed using SYBR Premix Ex Taq (Takara, #RR420) following the manufacturer's instructions. The expression levels of the indicated mRNAs were calculated using the 2^−ΔΔCt^ method and was normalized to internal control GAPDH. Chromatin immunoprecipitation (ChIP) assays were performed using SimpleChIP Enzymatic Chromatin IP Kit (magnetic beads) (Cell Signaling Technology, #9003S) according to the manufacturer's instructions. Quantitative results are displayed as corresponding fold change, and antirabbit IgG was used as a negative control. Primers used for qPCR and ChIP assays are listed in Tables S4 and S5.

### 2.6 Luciferase assays

Cells were transfected with 200 ng of pGL3 or pGL3‐PSG9 expression vector using Lipofectamine 2000. Renilla luciferase expression vector (pRL) (5 ng) was also transfected into cells as a transfection control. After 48 hours of transfection, luciferase assays were performed using a Dual‐Luciferase Reporter Assay System (Promega, #E1910) according to the manufacturer's instructions. The *PSG9* promoter activities were normalized to the corresponding values of the cotransfected Renilla luciferase gene.

### 2.7 Cell proliferation and colony formation assays

For cell counting kit‐8 (CCK‐8) assays, a total of 1 × 10^3^ cells were plated into 96‐well plates in triplicates and cultured for 5‐7 days. Total 10 μL CCK‐8 solution (Yeasen, #40203ES60) was added to each well. The plates were incubated in an incubator at 37°C for 3 hours, and then absorbance at 450 nm (A450) was determined. For colony formation assays, 1 × 10^3^ cells were plated into six‐well plates in triplicates and allowed to grow out for 10‐14 days. Cells were fixed by methanol, stained with 0.2% crystal violet solution and photographed. Colonies consisting of >50 cells were counted.

### 2.8 Wound‐healing and transwell migration and invasion assays

For wound‐healing assays, cells were plated into six‐well plates and allowed to grow to 90% confluence. Then, a 200 μL pipette tip was used to generate the wound. Cells were then cultured in serum‐free medium, and the wound healing was observed at the indicated times with inverted microscope, and photographs were taken. Transwell migration and invasion assays were performed using Boyden chambers with 8 μm pores (Corning Falcon, #353097) and Matrigel Invasion Chambers (Corning BioCoat, #354480), respectively, as described previously.^34^, ^35^ Medium containing 10% FBS in the lower chamber served as a chemoattractant. The migrated and invaded cells at the bottom of the inserts were fixed with methanol for 30 minutes and stained with 0.1% crystal violet for 1 hour at room temperature. Total number of cells in each chamber was counted. Cells were counted in 10 random fields under microscope.

### 2.9 Tumorigenicity and metastasis assays

All animal studies were approved by the Institutional Animal Care and Use Committee of Shanghai Cancer Center, Fudan University. For tumorigenesis assays, 2 × 10^6^ MDA‐MB‐231 cells stably expressing pCDH and HA‐PSG9 and LM2‐4175 cells stably expressing shNC and shPSG9 were injected into the mammary fat pad of 6‐week‐old BALB/c female mice (n = 6; State Key Laboratory of Oncogenes and Related Genes, Shanghai Cancer Institute, Shanghai, China). The mice were killed after 8 weeks of the inoculation, primary tumors were harvested, and tumor weight was determined. For experimental metastasis assays, 2 × 10^6^ MDA‐MB‐231 cells stably expressing pCDH and HA‐PSG9 as well as LM2‐4175 cells stably expressing shNC and shPSG9 in 200 μL of PBS were injected in the tail vein of 6‐week‐old BALB/c female nude mice (n = 6; State Key Laboratory of Oncogenes and Related Genes). After 6 weeks of injection, the lungs were excised, fixed in Bouin solution overnight, and lung colonies were counted under a Nikon SMZ1500 stereomicroscope (Nikon, Tokyo, Japan).

### 2.10 Clinical samples

All procedures were conducted in accordance with the Declaration of Helsinki and International Ethical Guidelines for Biomedical Research Involving Human Subjects, and approved by the institutional ethics review board of Fudan University Shanghai Cancer Center. All breast tumor samples and plasma samples were obtained from patients with breast cancer who underwent surgery at Fudan University Shanghai Cancer Center. These patients did not receive any therapies before surgical operation.

### 2.11 Enzyme‐linked immunosorbent assay (ELISA)

Peripheral blood samples were collected into vacuum tubes with EDTA anticoagulant and spun at 1000  ×  *g* for 10 min at 4°C. The plasma was stored at −80°C for further analysis. PSG9 concentrations in plasma samples were determined using PSG9 human ELISA kit (CUSABIO, #CSB‐EL018859HU) according to the manufacturer's instructions. All experiments were conducted in duplicate.

### 2.12 Immunohistochemical (IHC) staining

A total of 161 primary breast cancer specimens were obtained from the Department of Pathology, Fudan University Shanghai Cancer Center. IHC staining was performed as previously described,^36^ using a specific antibody against PSG9 (Novus, #NBP2‐19980) at a 1:150 dilution. No primary antibody or normal Rabbit IgG was used as a negative control. The representative photographs were taken using Olympus BX43 microscope. Interpretation of IHC results was performed by two independent pathologists who were blinded to the clinicopathological information. Slides were evaluated for a standard semiquantitative immunoreactive score (IRS) as described previously.^37^ By recording the percentage of positive staining (0 = negative, 1 ≤ 10%, 2 = 10‐50%, 3 ≥ 50%) and staining intensity (0 = no, 1 = weak, 2 = moderate, 3 = strong) for each sample, IRS (0‐9) was calculated by multiplying positive staining percentage with staining intensity.

### 2.13 Purification of recombinant proteins

The expression and purification of GST‐tagged or His‐tagged proteins were performed as described previously.^38^ The purified proteins were immediately used or frozen at −80°C for GST pull‐down assays as described previously.^38^

### 2.14 Statistical analysis

All data are presented as the mean ± standard deviation from at least three independent experiments. The unpaired two‐tailed Student's *t*‐test was used to compare data between two groups using SPSS20 (IBM, Armonk, NY). The probability of survival was estimated with the Kaplan‐Meier method and differences between groups were evaluated by the log‐rank test. *P*‐values of less than .05 were considered statistically significant.

## 3 RESULTS

### 3.1 PSG9 expression levels are elevated in tumor tissues and plasma specimens from breast cancer patients, and are associated with poor prognosis

To investigate the potential role of the members of PSG gene family in breast cancer progression, we first analyzed their mRNA levels in breast tumors and normal breast tissues using RNA‐sequencing data from The Cancer Genome Atlas (TCGA) and Genotype‐Tissue Expression (GTEx) databases.^27^ As shown in Figure S1, the mRNA levels of PSG9, but not other PSGs, were significantly upregulated in breast tumors relative to normal breast tissues. The noted upregulation of PSG9 mRNA levels in breast tumors was further confirmed in the Oncomine cancer microarray database (Finak Breast Dataset^39^) (Figure S2A). Analysis of the Gene Expression Omnibus (GEO) dataset (GSE25066)^40^ revealed that PSG9 levels were upregulated in the basal‐like subtype of breast tumors, the most aggressive subtype of breast cancer with poor prognosis, compared to the nonbasal type ones (Figure S2B). Moreover, breast tumors with distant metastasis expressed higher levels of PSG9 than those without metastasis (Figure S2C). In line with these results, survival analyses of multiple publicly available datasets, including E‐MTAB‐365,^41^ GSE9195,^42^ GSE25066,^40^ and GSE11121,^43^ revealed that high PSG9 expression was associated with poor relapse‐free survival and distant metastasis‐free survival of patients with breast cancer (Figure S3A,B). Together, these bioinformatic data indicate that dysregulation of PSG9 may contribute to breast cancer progression.

**FIGURE 1:**
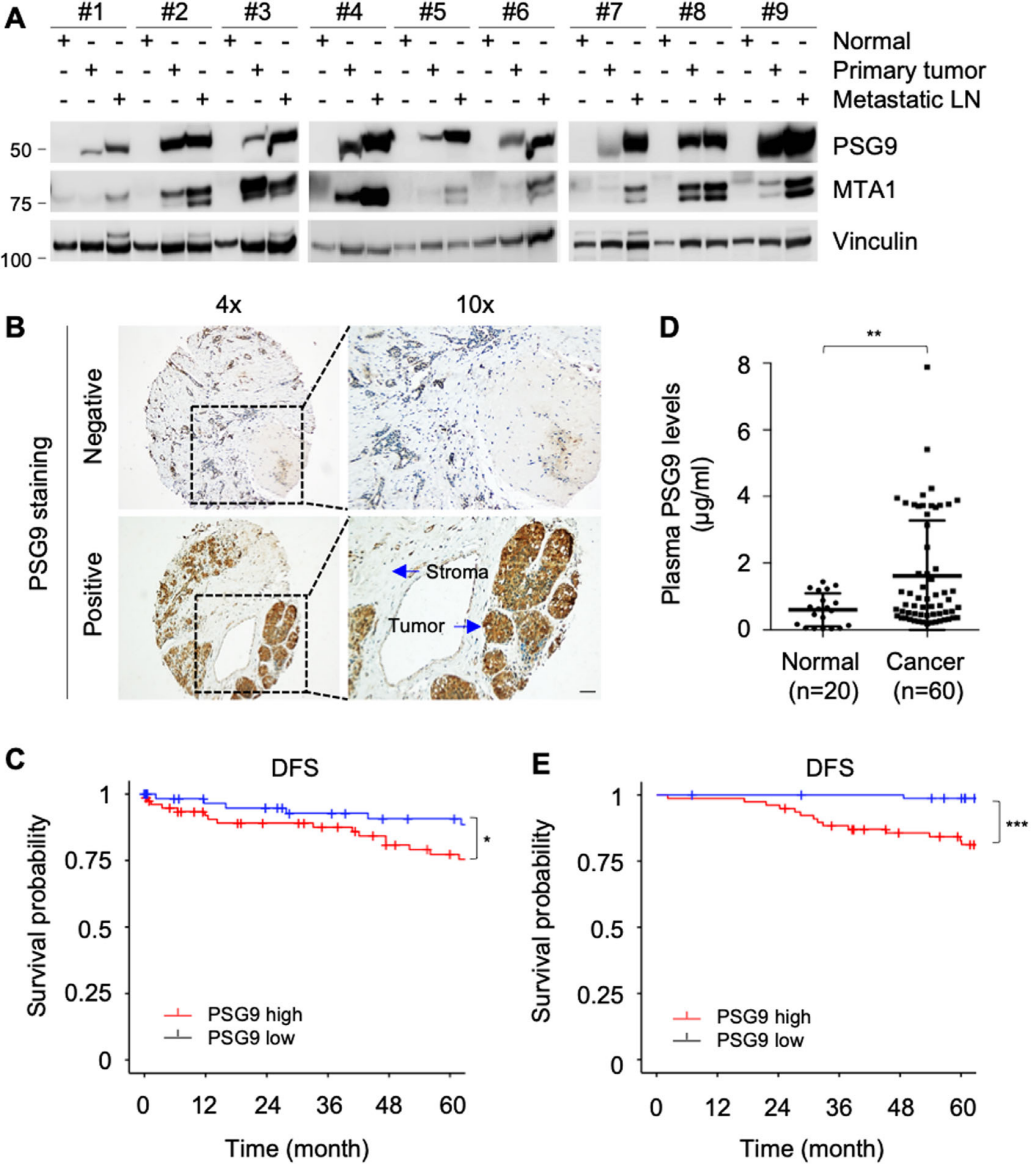
PSG9 levels are elevated in tumor tissues and plasma specimens from breast cancer patients and are associated with poor prognosis. A, Total lysates from nine pairs of matched adjacent normal breast tissues, primary breast tumors, and metastatic lymph node (LN) tissues were subjected to immunoblotting analysis with the indicated antibodies. Oncoprotein MTA1 was used as a positive control. B, Total 161 surgical specimens from breast cancer patients with clinical follow‐up information were subjected to immunohistochemical staining of PSG9 with a specific antibody against PSG9 (Novus, #NBP2‐19980). The representative images for PSG9 staining are shown. Bar, 25 μM. C, Kaplan‐Meier curves of disease‐free survival (DFS) of 161 breast cancer patients with high or low PSG9 expression based on the median of IRS value. Statistics analysis was performed by the log‐rank test. D, Detection of PSG9 levels by ELISA in plasma specimens from 20 healthy controls and 60 patients with breast cancer. E, Kaplan‐Meier curves of DFS of 161 breast cancer patients with high or low PSG9 plasma levels. *P < .05; **P < .01; ***P < .001

To validate these results, we examined PSG9 protein levels in nine pairs of matched adjacent normal breast tissues, primary breast tumors, and metastatic lymph node (LN) tissues by immunoblotting with an anti‐PSG9 antibody. Results showed that PSG9 levels were elevated in primary breast tumor tissues, particularly in metastatic LNs, compared to matched normal breast tissues (Figure 1A). Metastasis‐associated protein 1 (MTA1), a putative oncoprotein with pivotal roles in cancer progression and metastasis,^44^ was used as a positive control in these assays. To define the prognostic significance of PSG9 expression in breast cancer patients, we then performed IHC staining of PSG9 in 161 surgical specimens from patients diagnosed with invasive breast cancer with an anti‐PSG9 antibody. The representative images for PSG9 staining are shown in Figure 1B. Semiquantitative evaluation of PSG9 expression was performed using IRS on the basis of the intensity of PSG9 staining and the percentage of PSG9‐positive cells. According to the median of IRS value, 51.6% (83/161) and 48.4% (78/161) patients had low and high expression of PSG9, respectively. Survival analysis using Kaplan‐Meier curve showed that patients with high PSG9 expression had significantly shorter disease‐free survival (DFS) than those with low PSG9 expression (Figure 1C, *P *= .026). Moreover, subclassified analyses revealed that the expression levels of PSG9 were positively correlated with the presence of LN metastasis (*P *= .023) and with HER2 positivity (*P *= .036) (Table S6). In contrast, there was no significant correlation between PSG9 expression levels and other clinicopathological factors, including age, menopausal status, tumor grade, and ER/PR status in the case of the limited sample size (Table S6).

As PSG9 is a secreted glycoprotein, we next measured plasma levels of PSG9 in 20 healthy, nonpregnant females and 60 breast cancer patients by ELISA. As shown in Figure 1D, PSG9 levels in breast cancer patients were higher than those in normal subjects. To further explore the potential prognostic value of the plasma levels of PSG9, we detected PSG9 levels in plasma specimens from 161 breast cancer patients by ELISA. According to the median PSG9 level, these samples were divided into high‐ and low‐expression groups (77 *vs* 84 cases, respectively). As expected, high plasma PSG9 levels were associated with poor DFS of breast cancer patients (Figure 1E, *P *= .00043). In addition, plasma PSG9 levels were positively correlated with the presence of LN metastasis, but not other clinicopathological factors (Table S7). Collectively, both bioinformatic and experimental data suggest that PSG9 is upregulated in breast tumors and is associated with poor prognosis of breast cancer patients.

### 3.2 PSG9 promotes breast cancer cell proliferation and colony formation*in vitro* and tumor growth *in vivo*

**FIGURE 2:**
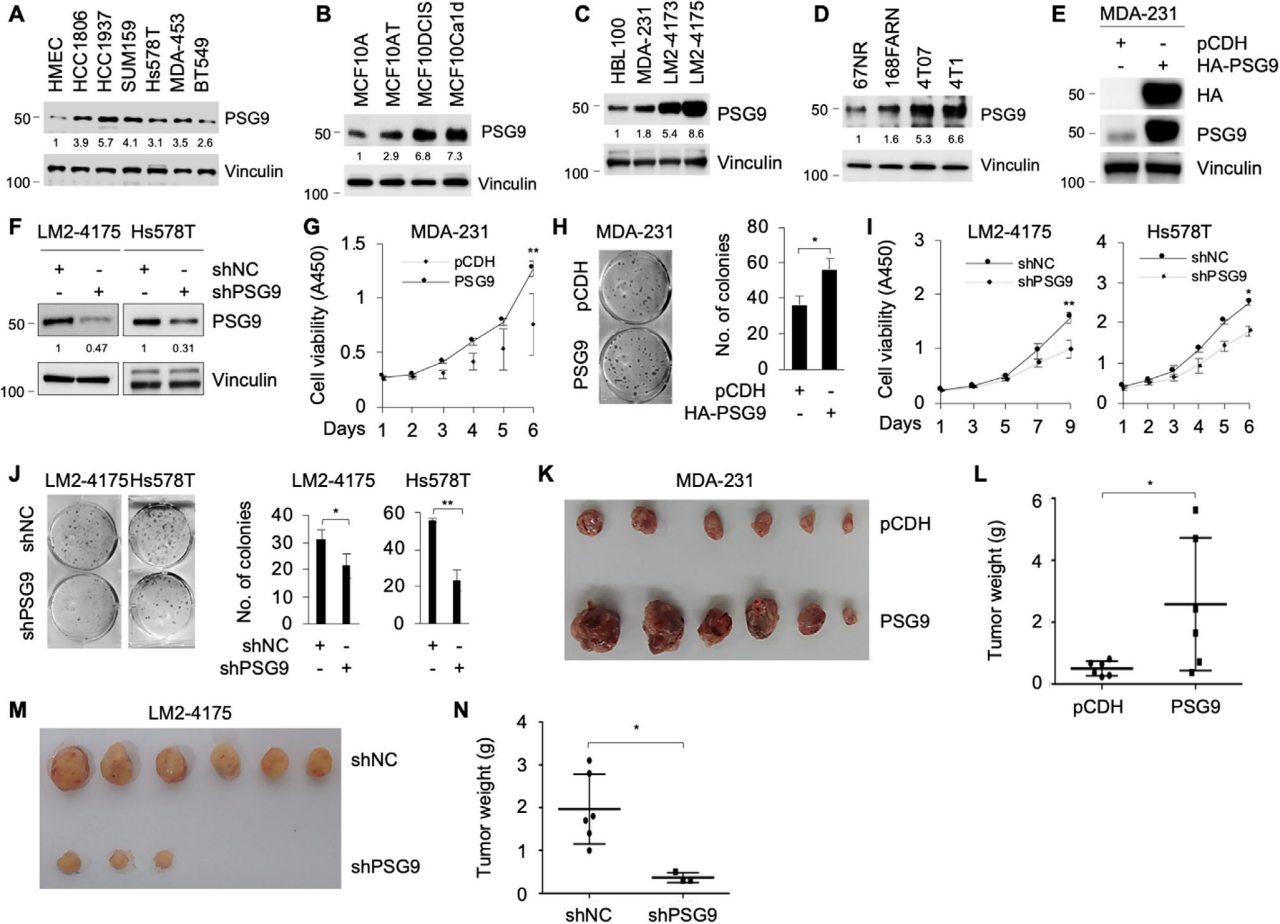
PSG9 promotes breast cancer cell proliferation and colony formation *in vitro* and tumor growth *in vivo*. A, Immunoblotting analysis of PSG9 expression levels in normal breast epithelial cell line HMEC and multiple breast cancer cell lines. The expression levels of PSG9 were normalized to those of vinculin. B‐D, Immunoblotting analysis of PSG9 expression levels in MCF10A series of cell lines (B), MDA‐231 sublines (C), and four isogenic murine breast cancer cell lines (D). The expression levels of PSG9 were normalized to those of vinculin. E, MDA‐231 cells stably expressing pCDH and HA‐PSG9 were subjected to immunoblotting analysis with the indicated antibodies. F, LM2‐4175 and Hs578T cells stably expressing shNC and shPSG9 were subjected to immunoblotting analysis with the indicated antibodies. The expression levels of PSG9 were normalized to those of vinculin. G and H, MDA‐MB‐231 cell stably expressing pCDH and HA‐PSG9 were subjected to CCK‐8 (G) and colony growth assays (H). Representative image of survival colonies (H, left) and quantitative results from three biological replicates (H, right). I and J, LM2‐4175 and Hs578T cells stably expressing shNC and shPSG9 were subjected to CCK‐8 (I) and colony growth assays (J). Representative image of survival colonies (J, left) and quantitative results from three biological replicates (J, right). K and L, MDA‐231 cells stably expressing pCDH and HA‐PSG9 were injected into mammary fat pads of 6‐week‐old female BALB/c nude mice (n = 6). After 8 weeks of being injected, xenograft tumors were harvested. Photographs of harvested tumors (K) and tumor weight (L) are shown. M and N, LM2‐4175 cells stably expressing shNC and shPSG9 were injected into mammary fat pads of 6‐week‐old female BALB/c nude mice (n = 6). After 8 weeks of being injected, xenograft tumors were harvested. Photographs of harvested tumors (M) and tumor weight (N) are shown. *P < .05; **P < .01

To determine the functional role of PSG9 in breast cancer progression, we first examined PSG9 expression levels by immunoblotting in normal HMEC and multiple breast cancer cell lines, including HCC1806, HCC1937, SUM159, Hs578T, MDA‐MB‐453 (MDA‐453), and BT549. Results showed that PSG9 was frequently upregulated in breast cancer cell lines compared to normal HMEC control (Figure 2A). Furthermore, immunoblotting analysis of PSG9 expression in three well‐documented isogenic cell line model systems for breast cancer progression, including MCF10A series of cell lines (nonmalignant MCF10A, premalignant MCF10AT, highly proliferative and locally invasive MCF10DCIS, and metastatic MCF10CA1d),^29^, ^30^, ^31^, ^32^ MDA‐MB‐231 sublines (MDA‐231 and its highly metastatic variants LM2‐4173 and LM2‐4175),^28^ and four murine breast cancer cell lines (nonmetastatic 67NR, weakly metastatic 168FARN and 4TO7, and highly metastatic 4T1),^33^ revealed that metastatic breast cancer cell lines expressed relatively higher levels of PSG9 than nonmetastatic ones (Figure 2B‐D). According to the relative expression levels of PSG9 in these cell lines and the tumorigenic and metastatic properties of these cell lines*in vivo* in light of subsequent mouse xenograft experiments, we stably expressed HA‐PSG9 in MDA‐231 cells and knocked down PSG9 in LM2‐4175 and Hs578T cells by infection with lentiviral vectors encoding HA‐PSG9 and shRNA targeting human PSG9 (shPSG9), respectively. The expression status of PSG9 in the resultant cell lines was verified by immunoblotting (Figure 2E,F). CCK‐8 and colony‐formation assays revealed that ectopic expression of PSG9 in MDA‐231 cells enhanced cell proliferation (Figure 2G) and generated more survival colonies (Figure 2H) as compared with empty vector pCDH control. In contrast, knockdown of PSG9 in LM2‐4175 and Hs578T cells significantly reduced cell proliferation (Figure 2I) and colony formation (Figure 2J).

To determine whether PSG9 contributes to breast cancer growth *in vivo*, MDA‐231 cells stably expressing pCDH and HA‐PSG9 were injected into the mammary fat pad of 6‐week‐old female BALB/c nude mice. Consistent with *in vitro* results, ectopic expression of PSG9 significantly promoted tumor growth in mice implanted with MDA‐231 cells stably expressing HA‐PSG9 as compared with mice implanted with pCDH expressing control cells (Figure 2K). The weight (Figure 2L) of the implanted tumors with PSG9 overexpression was significantly higher than that of the tumors expressing pCDH control. Conversely, knockdown of PSG9 in LM2‐4175 cells slowed down tumor growth than scramble controls, even no tumors were observed in some mice (Figure 2M,N). Together, these results suggest that PSG9 promotes breast cancer cell proliferation and colony formation *in vitro* and enhances xenograft tumor growth *in vivo*.

### 3.3 PSG9 enhances breast cancer cell migratory and invasive potential*in vitro* and lung colonization *in vivo*

**FIGURE 3:**
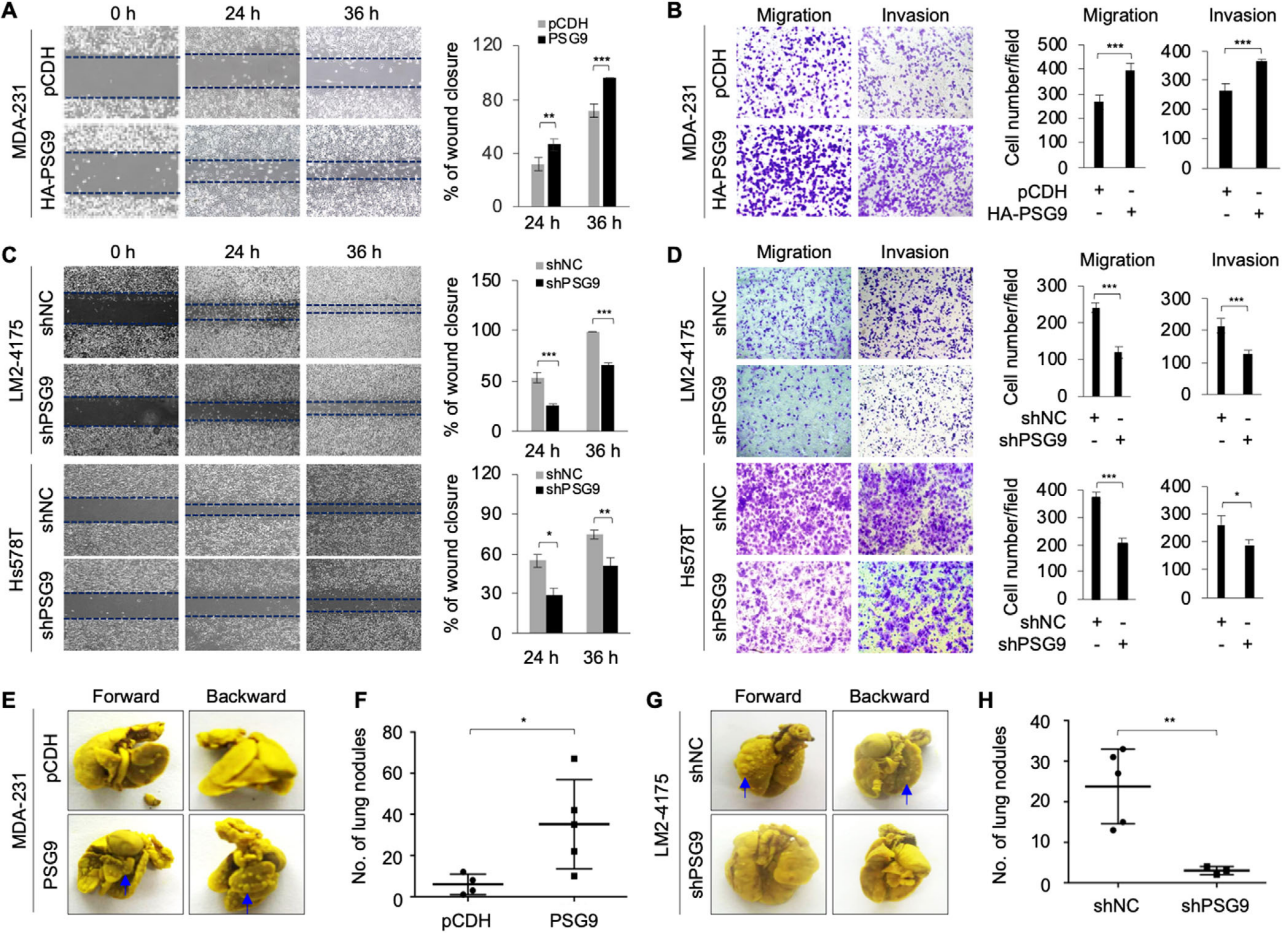
PSG9 enhances breast cancer cell migratory and invasive potential i*n vitro* and lung metastasis*in vivo*. A and B, MDA‐231 cells stably expressing pCDH and HA‐PSG9 were subjected to wound‐healing assays (A) or Boyden's chamber migration assays and Matrigel‐coated invasion assays (B). Representative images (A, left) and quantitative results of percent wound closure from three biological replicates (A, right). Representative images (B, left) and quantitative results of migrated and invaded cells from three biological replicates (B, right). C and D, LM2‐4175 and Hs578T cells stably expressing shNC and shPSG9 were subjected to wound‐healing assays (C) or Boyden's chamber migration assays and Matrigel‐coated invasion assays (D). Representative images (C, left) and quantitative results of percent wound closure from three biological replicates (C, right). Representative images (D, left) and quantitative results of migrated and invaded cells from three biological replicates (D, right) are shown. E and F, MDA‐231 cells stably expressing pCDH and HA‐PSG9 were injected into 6‐week‐old female BALB/c nude mice (n = 6) through the tail vein. After 6 weeks of injection, the lungs were harvested and stained with Bouin's solution. Representative images of lung metastasis (E) and quantitative results of lung nodules (F) are shown. G and H, LM2‐4175 cells stably expressing shNC and shPSG9 were injected into 6‐week‐old female BALB/c nude mice (n = 6) through the tail vein. After 6 weeks of injection, the lungs were harvested and stained with Bouin's solution. Representative images of lung metastasis (G) and quantitative results of lung nodules (H) are shown. *P < .05; **P < .01; ***P < .001

As a feature of breast cancer cells is their invasive and metastatic capacity,^2^ we next determined whether PSG9 could affect the migratory and invasive potential of breast cancer cells. Wound‐healing and transwell migration and invasion assays showed that ectopic expression of PSG9 in MDA‐MB‐231 cells enhanced cell migration and invasion compared to empty vector control (Figure 3A,B). In contrast, knockdown of PSG9 dramatically reduced the migratory and invasive capacity of LM2‐4175 and Hs578T cells (Figure 3C,D).

To examine whether PSG9 affects metastatic properties of breast cancer cells *in vivo*, MDA‐231 cells stably expressing pCDH and HA‐PSG9 were injected into the lateral tail vein of 6‐week‐old athymic nude mice. After 6 weeks of injection, overexpression of PSG9 dramatically increased the number of metastatic tumors in the lungs of mice (Figure 3E,F). To further validate these data, we repeated this set of experiments in a mouse model using PSG9‐depleted LM2‐4175 cells and corresponding control cells. Results showed that knockdown of PSG9 reduced the number of metastatic tumor nodes in the lungs of mice (Figure 3G,H). Together, these data confirm that PSG9 enhances migratory, invasive, and metastatic potential of breast cancer cells both *in vitro* and *in vivo*.

### 3.4 TGF‐β1 transcriptionally activates PSG9 through canonical TGFBR‐Smad pathway

**FIGURE 4:**
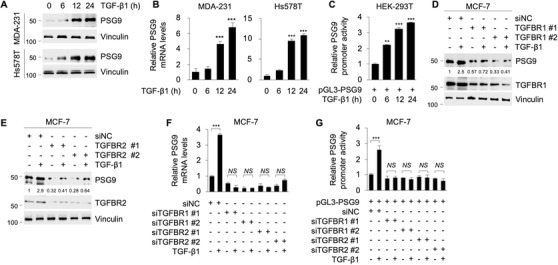
TGF‐β1 transcriptionally activates PSG9 in a TGF‐β receptor dependent manner. A and B, MDA‐231 and Hs578T cells were cultured in serum‐free media for 24 hours and then treated with or without 10 ng/mL TGF‐β1 for the indicated times. Immunoblotting (A) or qPCR (B) analyses were carried out to detect PSG9 expression levels. C, HEK293T cells were transfected with pGL3‐PSG9. After 24 hours of transfection, cells were cultured in serum‐free media for another 24 hours and then treated with or without 10 ng/mL TGF‐β1 for the indicated times. The *PSG9* promoter activity was determined using a Dual‐Luciferase Reporter Assay System and normalized to the values of Renilla luciferase. D‐F, MCF‐7 cells were transfected with negative control siRNA (siNC) or two independent siRNAs targeting TGFBR1 (siTGFBR1) or TGFBR2 (siTGFBR2). After 24 hours of transfection, cells were cultured in serum‐free media for 24 hours and then treated with or without 10 ng/mL of TGF‐β1 for another 24 hours. Immunoblotting analyses were carried out with the indicated antibodies (D and E). The expression levels of PSG9 were normalized to those of vinculin. qPCR analyses were carried out to detect PSG9 mRNA levels (F). G, MCF‐7 cells were transfected with siNC or two independent siRNAs targeting TGFBR1 (siTGFBR1) or TGFBR2 (siTGFBR2). After 24 hours of transfection, cells were transfected with pGL3‐PSG9 and cultured in serum‐free media for 24 hours, followed by treatment with or without 10 ng/mL of TGF‐β1 for another 24 hours. The *PSG9* promoter activity was determined using a Dual‐Luciferase Reporter Assay System and normalized to the values of Renilla luciferase. **P < .01; ***P < .001; NS, no significance

To address the molecular mechanisms underlying the noted upregulation and oncogenic role of PSG9 in breast cancer cells, we analyzed the promoter sequence of the human *PSG9* gene within 1‐kb region upstream from the transcriptional start site. Two putative SBEs (CAGACA)^45^, ^46^ in the *PSG9* promoter were found, located at −996 to −991 and −95 to −90 (Figure S4A), indicating that PSG9 may be regulated by the TGF‐β/Smad signaling pathway. To test this notion, MDA‐231 and Hs578T cells were treated with or without 10 ng/mL TGF‐β1, the most abundant and ubiquitously expressed TGF‐β isoform, for the indicated times, and the expression levels of PSG9 were evaluated by immunoblotting and qPCR analyses. Results showed that TGF‐β1 treatment induced PSG9 expression at both protein (Figure 4A) and mRNA (Figure 4B) levels in a time‐dependent manner. Moreover, treatment of MDA‐231 and Hs578T cells with SB431542, a TGFBR1 (ALK5) inhibitor,^47^ attenuated TGF‐β1‐mediated PSG9 induction (Figure S4B,C). Upregulation of PSG9 mRNA and protein levels following TGF‐β1 treatment was also observed in MCF‐7 cells (Figure S4D,E). To examine whether *PSG9* promoter activities are affected by the TGFβ/Smad pathway, HEK293T cells were transfected with a luciferase reporter construct encoding 1‐kb region of the *PSG9* promoter upstream of transcription start site (pGL3‐PSG9), and then treated with or without 10 ng/mL TGF‐β1 for the indicated times. Luciferase reporter assays revealed that TGF‐β1 significantly enhanced the activity of the *PSG9* promoter in a time‐dependent manner (Figure 4C). These data suggest that PSG9 is a transcriptional target of the TGF‐β1 signaling pathway.

**FIGURE 5:**
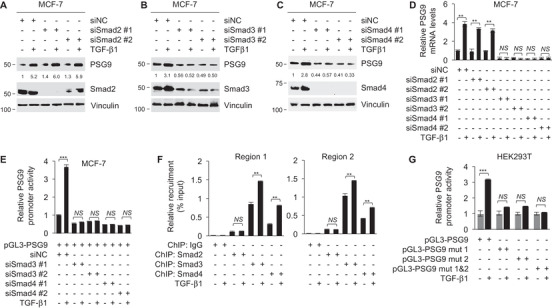
TGF‐β1 transcriptionally activates PSG9 by enhancing recruitment of Smad3 and Smad4 onto the *PSG9* promoter regions containing two putative Smad‐binding elements (SBEs). A‐D, MCF‐7 cells were transfected with siNC or two independent siRNAs targeting Smad2 (siSmad2), Smad3 (siSmad3), or Smad4 (siSmad4). After 24 hours of transfection, cells were cultured in serum‐free media for 24 hours and then treated with or without 10 ng/mL of TGF‐β1 for another 24 hours. Immunoblotting analyses were carried out with the indicated antibodies (A‐C). The expression levels of PSG9 were normalized to those of vinculin. qPCR analyses were carried out to detect PSG9 mRNA levels (D). E, MCF‐7 cells were transfected with siNC, siSmad3, or siSmad4. After 24 hours of transfection, cells were transfected with pGL3‐PSG9 and cultured in serum‐free media for 24 hours, followed by treatment with or without 10 ng/mL of TGF‐β1 for another 24 hours. The *PSG9* promoter activity was determined using a Dual‐Luciferase Reporter Assay System and normalized to the values of Renilla luciferase. F, MCF‐7 cells were cultured in serum‐free media for 24 hours and treated with or without 10 ng/mL of TGF‐β1 for another 24 hours. ChIP assays were performed with control IgG or a specific antibody against Smad2, Smad3, or Smad4, followed by qPCR analysis with specific primers amplifying *PSG9* promoter regions (region 1 and region 2) containing two putative Smad‐binding elements (SBEs). Recruitment of Smad3 and Smad4 to the *PSG9* promoter regions containing two putative SBEs was normalized to the Input. G, MCF‐7 cells were transfected with pGL3, pGL3‐PSG9, or mutant pGL3‐PSG9 and cultured in serum‐free media for 24 hours, followed by treatment with or without 10 ng/mL of TGF‐β1 for another 24 hours. The *PSG9* promoter activity was determined using a Dual‐Luciferase Reporter Assay System and normalized to the values of Renilla luciferase. Note: mut1, the first SBE was mutated; mut2, the second SBE was mutated; mut 1&2, both SBEs were mutated. **P < .01; ***P < .001; NS, no significance

TGF‐β1 transmits signals mainly through two transmembrane receptors (TGFBR1/2) and three Smad proteins (Smad2/3/4).^50^, ^56^ To provide further mechanistic insights into TGF‐β1‐induced PSG9 expression, we first knocked down endogenous TGFBR1 or TGFBR2 using two independent siRNAs in MCF‐7 cells. The reason for using this cell line for siRNA transfection is that it has higher transfection efficiency than MDA‐231 and Hs578T cells do. Transfection of siRNAs targeting TGFBR1 (siTGFBR1) or TGFBR2 (siTGFBR2) significantly impaired TGF‐β1‐induced upregulation of PSG9 protein and mRNA levels and promoter activities (Figure 4D‐G). Moreover, siRNA‐mediated knockdown of Smad3 or Smad4, but not Smad2, hindered the upregulation of PSG9 protein and mRNA levels induced by TGF‐β1 (Figure 5A‐D). These results suggest that Smad3 and Smad4 have a more dominant role than Smad2 in TGF‐β1‐induced transcriptional activation of PSG9. Luciferase reporter assays further demonstrated that knockdown of Smad3 or Smad4 attenuated TGF‐β1‐induced *PSG9* promoter activities (Figure 5E). ChIP assays with an anti‐Smad2, anti‐Smad3, or anti‐Smad4 antibody revealed that TGF‐β1 enhanced the binding of Smad3 and Smad4, but not Smad2, to the promoter regions of *PSG9* containing two putative SBEs (Figure 5F). Furthermore, mutation of one of those two SBEs in *PSG9* promoter significantly reduced TGF‐β1‐induced upregulation of *PSG9* promoter activities, and the promoter activities of *PSG9* induced by TGF‐β1 were completely abrogated when both SBEs were mutated (Figure 5G). These results indicate that both SBEs in the *PSG9* promoter are necessary for TGF‐β1‐induced transcriptional activation of PSG9. Taken together, these findings suggest that TGF‐β1 transcriptionally activates PSG9 through the canonical TGFBR‐Smad pathway.

### 3.5 PSG9 is essential for TGF‐β1‐induced EMT and breast cancer cell migration and invasion

**FIGURE 6:**
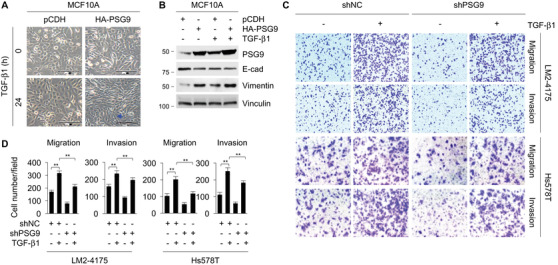
PSG9 contributes to TGF‐β1‐induced epithelial‐mesenchymal transition (EMT) and breast cancer cell migration and invasion. A, MCF10A cells stably expressing pCDH and HA‐PSG9 were cultured in serum‐free media for 24 hours, treated with or without 10 ng/mL of TGF‐β1 for another 24 hours, and photographed under a phase contrast microscopy. Scale bar: 100 μM. B, MCF10A cells stably expressing pCDH and HA‐PSG9 were cultured in serum‐free media for 24 hours, treated with or without 10 ng/mL of TGF‐β1 for another 24 hours, and then subjected to immunoblotting analysis with the indicated antibodies. C and D, LM2‐4175 and Hs578T cells stably expressing shNC and shPSG9 were cultured in serum‐free media for 24 hours and treated with or without 10 ng/mL of TGF‐β1 for another 24 hours, and then subjected to Boyden's chamber migration assays and Matrigel‐coated invasion assays. Representative images (C) and quantitative results of migrated and invaded cells from three biological replicates (D) are shown. **P < .01

TGF‐β is a potent inducer of EMT both during development and in cancer. To understand the role of PSG9 in TGF‐β1‐induced EMT, we stably expressed HA‐PSG9 in human mammary epithelial cell line MCF10A, a commonly used cell model for TGF‐β‐induced EMT.^48^, ^49^ Treatment with TGF‐β1 resulted in a fibroblast‐like cell elongation in control cells, and ectopic expression of PSG9 conferred a more pronounced mesenchymal phenotype, evidenced by more elongated cells (Figure 6A). Immunoblotting assays showed that ectopic expression of PSG9 in MCF10A cells led to a decrease in the expression of epithelial cell marker E‐cadherin (E‐cad), accompanied by an increase in the expression of mesenchymal cell marker vimentin, and that TGF‐β1 treatment enhanced the effect of PSG9 on vimentin expression (Figure 6B). These results suggest that PSG9 plays an important role in TGFβ‐induced EMT. In support of this notion, transwell migration and invasion assays demonstrated that treatment with TGF‐β1 enhanced the migratory and invasive ability of LM2‐4175 and Hs578T cells, but the noted effect of TGF‐β1 on breast cancer cell migration and invasion was compromised following knockdown of endogenous PSG9 in both cell lines (Figure 6C,D). These results highlight that PSG9 contributes to the TGF‐β1‐induced EMT and breast cancer cell migration and invasion.

### 3.6 PSG9 enhances stability of Smad2, Smad3, and Smad4 proteins and regulates expression of target genes of TGF‐β/Smad signaling

**FIGURE 7:**
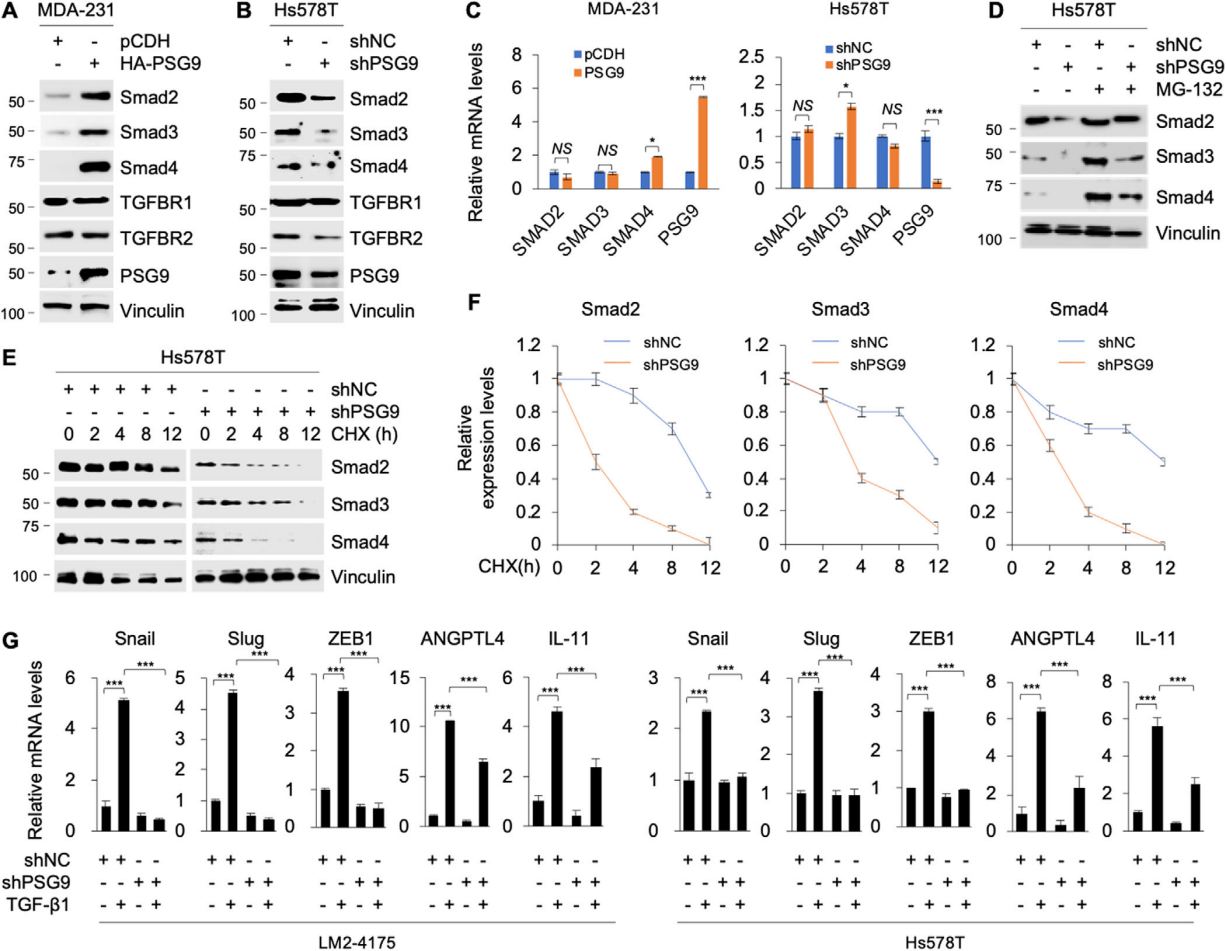
PSG9 enhances the stability of Smad2, Smad3, and Smad4 proteins and regulates expression of target genes of TGF‐β/Smad signaling. A and B, MDA‐231 cells stably expressing pCDH and HA‐PSG9 (A) and Hs578T cells stably expressing shNC and shPSG9 (B) were subjected to immunoblotting analysis with the indicated antibodies. C, MDA‐231 cells stably expressing pCDH and HA‐PSG9 (left) and Hs578T cells stably expressing shNC and shPSG9 (right) were subjected to qPCR analysis of Smad2/3/4 and PSG9 mRNA levels. D, Hs578T cells stably expressing shNC and shPSG9 were treated with or without 10 μM MG‐132 for 6 hours, and then subjected to immunoblotting with the indicated antibodies. E and F, Hs578T cells stably expressing shNC and shPSG9 were treated with or without 100 μg/mL of cycloheximide (CHX) for the indicated times, and then analyzed by immunoblotting with the indicated antibodies (E). The expression levels of Smad2, Smad3, or Smad4 were normalized to those of vinculin, and quantitative results from three biological replicates are shown (F). G, LM2‐4175 and Hs578T cells stably expressing shNC and shPSG9 were cultured in serum‐free media for 24 hours, treated with or without 10 ng/mL of TGF‐β1 for another 24 hours, and then subjected to qPCR analysis to detect the expression levels of the indicated genes. **P < .01; ***P < .001

To further explore the impact of PSG9 on TGF‐β signaling, we next examined the protein expression levels of the key components of the TGF‐β signaling, including TGFBR1/2 and Smad2/3/4 in MDA‐231 cells stably expressing HA‐PSG9 by immunoblotting. Results showed that ectopic expression of PSG9 resulted in a significant increase in the protein levels of Smad2/3/4 but not TGFBR1/2 (Figure 7A). Conversely, knockdown of PSG9 by shRNA in Hs578T cells reduced the protein levels of Smad2/3/4 but not TGFBR1 (Figure 7B). In addition, we noticed that knockdown of PSG9 in Hs578T cells slightly downregulated the protein levels of TGFBR2. To investigate whether PSG9 affects the activation of the canonical TGF‐β/Smad signaling, we examined the phosphorylation status of Smad2/3 and the nuclear translocation of Smad2/3/4 in PSG9‐depleted LM2‐4175 and Hs578T cells after treatment with TGF‐β for 6 hours by immunofluorescence staining. Results showed that knockdown of PSG9 attenuated the levels of phosphorylated Smad2/3 and the nuclear translocation of Smad/2/3/4 in response to TGF‐β1 (Figure S5). In contrast, qPCR analysis revealed that overexpression or knockdown of PSG9 had no significant effect on the mRNA levels of Smad2/3/4 (Figure 7C). These results indicate that PSG9 regulates Smad2/3/4 at posttranscriptional level. In support of this notion, decreased Smad2/3/4 protein levels in PSG9‐depleted Hs578T cells were effectively restored following treatment with proteasome inhibitor MG‐132 (Figure 7D, compare lane 4 with 2). Furthermore, cycloheximide chase assays revealed that knockdown of PSG9 in Hs578T cells decreased the half‐lives of those three proteins (Figure 7E,F). These results indicate that PSG9 enhances the stability of Smad2/3/4.

To address the possible mechanism for the above observations, we next examined the subcellular localization of PSG9 by immunofluorescent staining. Results showed that PSG9 was localized in both cytoplasm and nucleus (Figure S6A). In agreement with our observations, another study recently reported that PSG9 is distributed in the cell membrane, cytoplasm, and nucleus of human umbilical vein endothelial cells and colorectal cancer cells.^50^ To examine whether PSG9 interacts with Smad2/3/4, we cotransfected Flag‐Smad2/3/4 and HA‐PSG9 into HEK293T cells and then carried out IP assays with an anti‐Flag antibody. Immunoblotting analyses with the indicated antibodies revealed PSG9 interacted with Smad2/3/4 (Figure S6B). GST pull‐down assays further demonstrated that GST‐Smad2/3/4 bound to His‐PSG9 (Figure S6C). These results indicate that PSG9 could directly interact with Smad2/3/4. Emerging evidence has shown that the stability of Smad proteins is regulated by ubiquitination. To investigate whether PSG9 could affect the ubiquitination of Smad2/3/4, HEK293T cells were transfected with Flag‐Smad2/3/4, V5‐ubiquitin, and HA‐PSG9 alone or in combination. The sequential IP and immunoblotting analyses with the indicated antibodies showed a significant decrease of poly‐ubiquitynated Smad2/3/4 proteins in PSG9 transfected cells (Figure S7A,B). These results suggest that PSG9 stabilizes Smad2/3/4 through blocking their proteasomal degradation. TGF‐β signaling contributes to breast cancer progression through, at least in part, activating Smad‐dependent transcriptional response. We further demonstrated that knockdown of PSG9 in LM2‐4175 and Hs578T cells compromised the expression levels of TGF‐β1/Smad target genes, including EMT‐related transcription factors Snail, Slug, ZEB1,^51^ angiopoietin‐like 4 (ANGPTL4),^52^ and osteolytic cytokine interleukin 11 (IL‐11)^53^ (Figure 7G).

## 4 DISCUSSION

**FIGURE 8:**
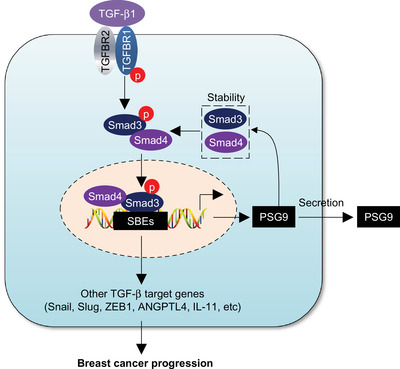
The proposed working model. TGF‐β1 transcriptionally activates PSG9 expression through the canonical TGF‐β receptor‐Smad pathway. In turn, PSG9 enhances the stability of Smad2, Smad3, and Smad4 by blocking their proteasomal degradation. As a result, PSP9 contributes to TGF‐β1‐induced epithelial‐mesenchymal transition, breast cancer cell migration and invasion, and the expression levels of TGF‐β target genes

In this study, we uncovered several interesting findings concerning the emerging role of PSG9 in breast cancer progression (Figure 8). First, PSG9 is upregulated in tumor tissues and plasma samples from breast cancer patients and its high expression is associated with poor prognosis. As the PSG genes belong to a subgroup of the carcinoembryonic antigen gene family, deregulation of PSG expression has been documented in some types of human cancer.^54^, ^55^ In this context, very limited evidence has shown that PSG9 protein levels are significantly increased in hepatocellular carcinoma and colorectal cancer patients and could serve as an indicator of patient prognosis.^50^, ^56^, ^57^, ^58^, ^59^ In this study, we provide the first evidence that PSG9 expression level was upregulated in breast tumors and metastatic LNs and was associated with shorter patient survival (Figure 1A‐C). Moreover, plasma PSG9 levels were also elevated in breast cancer patients and were associated with poor clinical outcome (Figure 1D,E).

Second, PSG9 is a transcriptional target of the canonical TGF‐β1/Smad pathway. The presence of two putative SBEs in PSG9 gene promoter prompted us to address whether PSG9 is regulated by TGF‐β1. Evidence presented here showed that TGF‐β1 induces PSG9 expression through enhancing the recruitment of Smad3 and Smad4 to the *PSG9* promoter regions containing SBEs (Figures 4 and 5). Importantly, mutation of those two SBEs present in the *PSG9* promoter, or knockdown of TGFBR1, TGFBR2, Smad 3, or Smad4 abolished the ability of TGF‐β1 to induce PSG9 expression (Figures 4 and 5). In contrast, depletion of Smad2 has no significant effect on TGF‐β1‐induced PSG9 expression. In support of our findings, it has been documented that Smad3 and Smad4, but not Smad2, are required for transcriptional regulation of several TGF‐β target genes, such as plasminogen activator inhibitor‐type 1,^60^ Smad7,^61^ collagen type VII α1 chain,^62^ inhibitor of DNA binding 1,^63^ tenascin‐C,^64^ and selenoprotein P.^65^ In addition, although a recent study documented that PSG1 expression correlates with the expression of TGF‐β in cervical cancer tissues,^66^ no information is currently available about whether PSG9 induces secretion and activation of TGF‐β1 in human cancer cells. Thus, whether PSG9 in turn induces secretion and activation of TGF‐β1 in breast cancer cells remains to be addressed in the future studies.

Third, PSG9 enhances the stability of Smad2, Smad3, and Smad4 proteins, which are key mediators of TGF‐β signaling. Like many key regulatory proteins, the activity and stability of Smad proteins are regulated by diverse posttranslational modifications, such as ubiquitination.^67^, ^68^ In this context, several ubiquitin ligases have been implicated in proteasomal degradation of Smad2 and Smad3, including Smad‐specific E3 ubiquitin protein ligase 2 (Smurf2),^69^ WW domain containing E3 ubiquitin protein ligase 1 (WWP1),^70^ NEDD4 like E3 ubiquitin protein ligase (Nedd4L)^71^ for Smad2 degradation, and RING‐box protein 1 (RBX1)^72^ and RING‐type E3 ubiquitin transferase CHIP^73^ for Smad3 degradation. In addition, Smad4 protein stability is regulated by ubiquitin ligase SCF β‐TrCP1.^74^ Jab1, also known as the subunit‐5 (CSN5) of COP9 signalosome, has been shown to interact directly with Smad4 and to induce its ubiquitin‐dependent degradation.^75^ In this study, we report that PSG9 enhances the stability of Smad2/3/4 through blocking their proteasomal degradation (Figure 7 and Figure S7). Given that PSG9 is a not a putative E3 ubiquitin ligase or deubiquitinase, we speculated that PSG9 suppresses the ubiquitination of Smad2/3/4 probably through affecting the binding of Smads2/3/4 with certain identified and/or unidentified enzymes related to the ubiquitination machinery. In support of this notion, Pin1, a peptidylprolyl cis‐trans isomerase, has been shown to promote Smad2/3 ubiquitination and degradation through enhancing the interaction of Smurf2, a Smad ubiquitin ligase, with Smad2/3.^76^

Fourth, both *in vitro* and *in vivo* functional assays demonstrated that PSG9 promotes tumor growth and metastasis (Figures 2 and 3). It is generally accepted that TGF‐β functions as a tumor suppressor in premalignant cells but as a tumor promoter in cancer cells,^77^ and that the switch between the antioncogenic and oncogenic properties of TGF‐β during cancer progression involves both cell‐intrinsic and environment‐mediated mechanisms.^78^ In this study, we demonstrated that PSG9 is involved in TGF‐β1‐induced EMT and breast cancer cell migration and invasion (Figure 6). In addition, PSG9 is involved in regulating the expression of TGF‐β target genes, including Snail, Slug, ZEB1, ANGPTL4, and IL‐11 (Figure 7 ). Snail, Slug, and ZEB1 are well‐established EMT‐related master transcription factors, which mediate the effects of TGF‐β through, at least in part, repression of E‐cad and initiation of EMT.^51^ ANGPTL4 mediates TGF‐β priming for mammary tumor dissemination to the lungs.^52^ IL‐11 is implicated in bone metastasis of breast cancer.^53^ These results support the notion that PSG9 promotes breast cancer metastasis.

One drawback of this story is that it remains unexplored how PSG9 promotes breast cancer cell proliferation and tumor growth. As PSG9 enhances the stability of Smad2/3/4 (Figure 7), there are two possibilities for the contribution of PSG9 to breast cancer cell proliferation and tumor growth. First, the stabilized Smad3 by PSG9 could regulate breast cancer cell proliferation and tumor growth. In this context, it has been shown that overexpression of Smad3 suppresses cell apoptosis and promotes cell proliferation, whereas treatment with Smad3 inhibitor (SIS3) promotes apoptosis and inhibits proliferation of breast cancer cells.^79^ Smad3 deficiency reduces growth and invasion capacity of triple‐negative breast cancer cells in comparison to Smad2, which has no effect.^80^ In contrast, Smad4 mainly contributes to breast cancer bone metastasis formation through transcriptional activation of IL‐11, a gene implicated in bone metastasis.^9^, ^53^ Second, PSG9 may potentiate TGF‐β‐induced Smad‐mediated transcriptional response to regulate breast cancer cell proliferation. Previous studies have demonstrated that the TGF‐β‐Smad pathway contributes to cancer cell proliferation and growth through transcriptional regulation of its downstream target genes. For example, it was reported by Bruna et al that the TGF‐β1‐Smad pathway promotes proliferation of human gliomas cells through inducing PDGF‐B gene expression.^81^ Similarly, González‐González et al found that transcription factor ATF4 acts as an active downstream target of the TGF‐β1‐Smad pathway to promote the growth and metastasis of triple‐negative breast cancer.^82^

In summary, findings presented here underscore the pivotal role of PSG9 in breast cancer progression and canonical TGF‐β/Smad pathway. As PSG9 is a secreted glycoprotein, it is interesting to carry out a population‐based cohort study to examine the possibility of PSG9 as a potential plasma biomarker for the prediction and/or prognosis of breast cancer.

### ETHICS STATEMENT

This study was approved by the institutional ethics review board of Fudan University Shanghai Cancer Center and by the Institutional Animal Care and Use Committee of Fudan University Shanghai Cancer Center.

## CONFLICT OF INTEREST

The authors declare that there is no conflict of interest.

## AUTHOR CONTRIBUTIONS

Ying‐Ying Liu and Sa Zhang performed the experiments. Tian‐Jian Yu, Fang‐Lin Zhang, Fan Yang, Yan‐Ni Huang, and Ding Ma contributed to data analysis. Guang‐Yu Liu, Zhi‐Ming Shao, and Da‐Qiang Li conceived the project and supervised the study. Ying‐Ying Liu and Da‐Qiang Li wrote the paper with inputs from all the authors.

## Supporting information


**Figure S1** PSG9 mRNA levels are elevated in breast tumors
**Figure S2** High expression of PSG9 correlates with an aggressive phenotype of breast cancer
**Figure S3** High expression of PSG9 is associated with poor prognosis of breast cancer patients
**Figure S4** PSG9 is a transcriptional target of TGF‐β1
**Figure S5** The effect of PSG9 on activation of the Smad signaling in response to TGF‐ꞵ1
**Figure S6** The subcellular localization of PSG9 and the interaction between PSG9 and Smad2/3/4
**Figure S7** The effect of PSG9 on the ubiquitination of Smad2/3/4
**Table S1** Primers used for molecular cloning of expression vectors through blocking their proteasomal degradation
**Table S2** siRNA target sequences
**Table S3** Antibodies used in this study
**Table S4** Primers for qPCR analysis
**Table S5** Primers for ChIP‐qPCR analysis
**Table S6** Characterization of clinicopathological features of 161 patients with primary breast cancer (IHC staining of PSG9)
**Table S7** Characterization of clinicopathological features of 161 patients with primary breast cancer (detection of plasma PSG9 levels by ELISA)

## Data Availability

All data generated or analyzed during this study are included in this article.
