# Supplementary material for: Pregnancy‐specific glycoprotein 9 acts as both a transcriptional target and a regulator of the canonical TGF‐β/Smad signaling to drive breast cancer progression
Source: Clin Transl Med. 2020 Dec 12;10(8):e245. doi: 10.1002/ctm2.245 (PMC7733318; doi:10.1002/ctm2.245)
Supplement: Supplementary file 1 — Figure S1 PSG9 mRNA levels are elevated in breast tumors Figure S2 High expression of PSG9 correlates with an aggressive phenotype of breast cancer Figure S3 High expression of PSG9 is associated with poor prognosis of breast cancer patients Figure S4 PSG9 is a transcriptional target of TGF‐β1 Figure S5 The effect of PSG9 on activation of the Smad signaling in response to TGF‐ꞵ1 Figure S6 The subcellular localization of PSG9 and the interaction between PSG9 and Smad2/3/4 Figure S7 The effect of PSG9 on the ubiquitination of Smad2/3/4 Table S1 Primers used for molecular cloning of expression vectors through blocking their proteasomal degradation Table S2 siRNA target sequences Table S3 Antibodies used in this study Table S4 Primers for qPCR analysis Table S5 Primers for ChIP‐qPCR analysis Table S6 Characterization of clinicopathological features of 161 patients with primary breast cancer (IHC staining of PSG9) Table S7 Characterization of clinicopathological features of 161 patients with primary breast cancer (detection of plasma PSG9 levels by ELISA) [file CTM2-10-e245-s001.pdf]

Supplementary Information for

Liu et al. Pregnancy-specific glycoprotein 9 acts as both a transcriptional target and a regulator of the canonical TGF-β/Smad signaling to drive breast cancer progression

The Supplementary Information includes

1. Supplementary Figures S1-S7
2. Supplementary Figure legends
3. Supplementary Tables S1-S7

Supplementary Figures and Figure legends

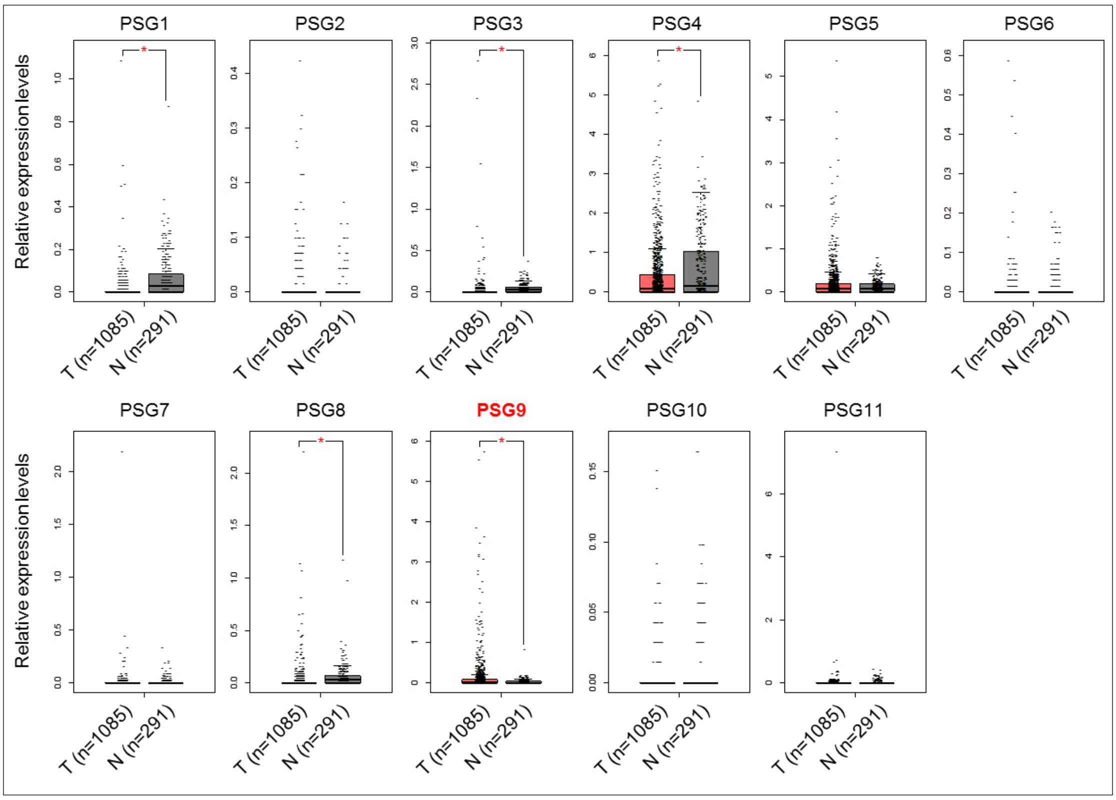

Figure S1. PSG9 mRNA levels are elevated in breast tumors

Analysis of mRNA levels of the members of PSG gene family in breast tumors and normal breast tissues using RNA-sequencing data from The Cancer Genome Atlas (TCGA) and Genotype-Tissue Expression (GTEx) databases. *$p < 0.05$.

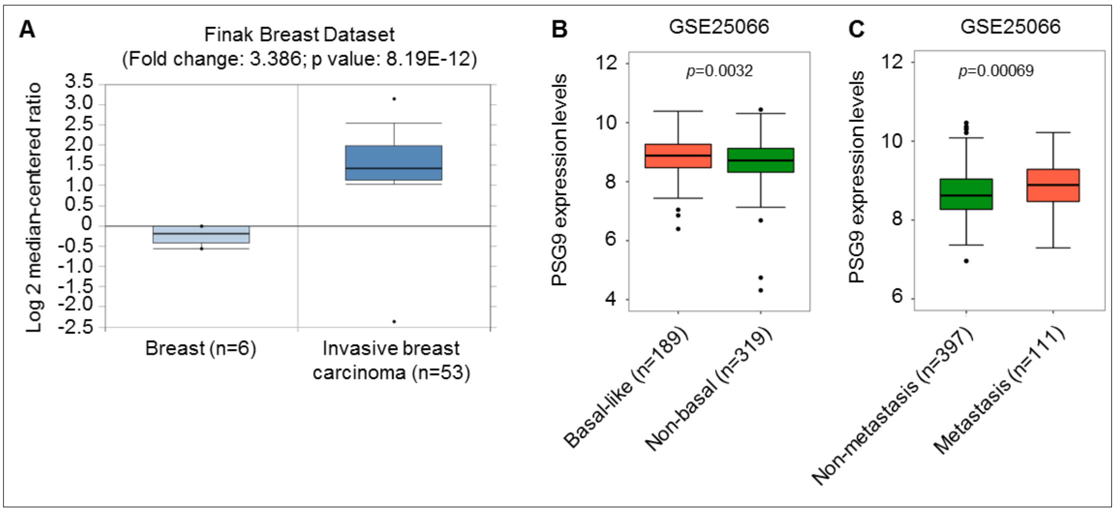

Figure S2. High expression of PSG9 correlates with an aggressive phenotype of breast cancer

(A) Analysis of PSG9 mRNA levels in breast tumors and normal breast tissues using the Oncomine cancer microarray database (Finak Breast Dataset [1]). (**B-C**) Analysis of PSG9 mRNA levels using the Gene Expression Omnibus (GEO) dataset (GSE25066). Box-plots indicate PSG9 mRNA levels in breast tumor according to molecular subtypes (B) and status of metastasis (C).

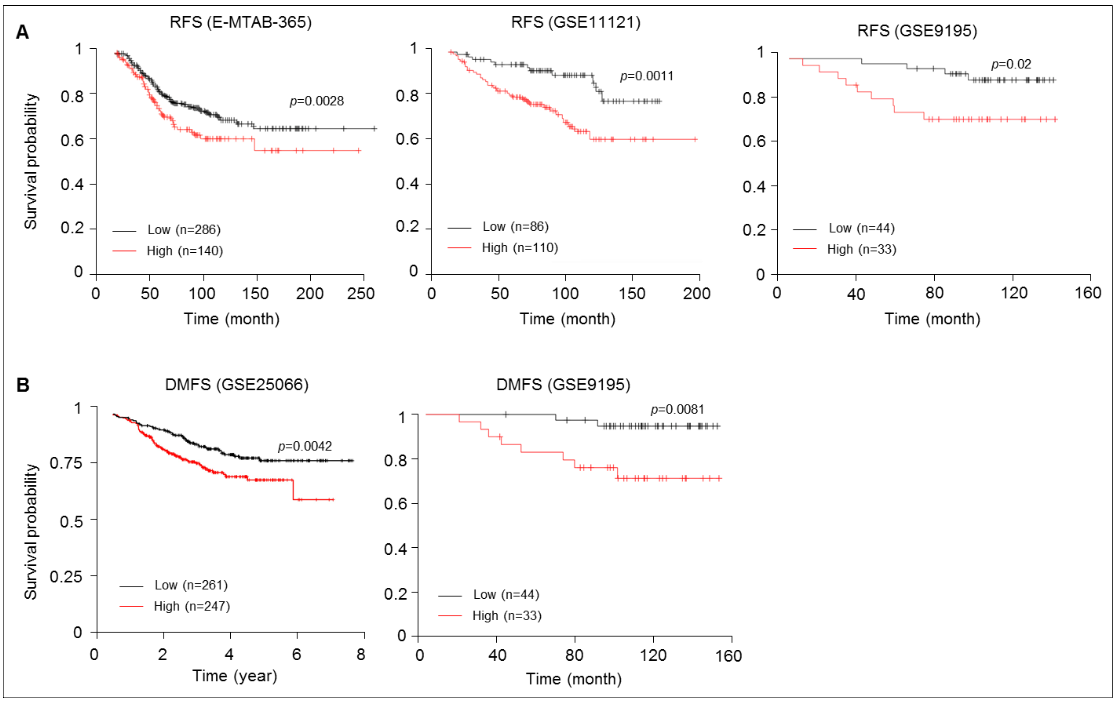

Figure S3. High expression of PSG9 is associated with poor prognosis of breast cancer patients

(**A**) Kaplan-Meier survival analysis for relapse-free survival (RFS) of breast cancer patients in E-MTAB-365 (left), GSE11121 (middle), GSE9195 (right) datasets according to PSG9 expression status. The *p* value is determined using the log-rank test. (**B**) Kaplan-Meier survival analysis for distant metastasis-free survival (DMFS) of breast cancer patients in GSE25066 and GSE9195 datasets according to PSG9 expression status. The *p* value is determined using the log-rank test.

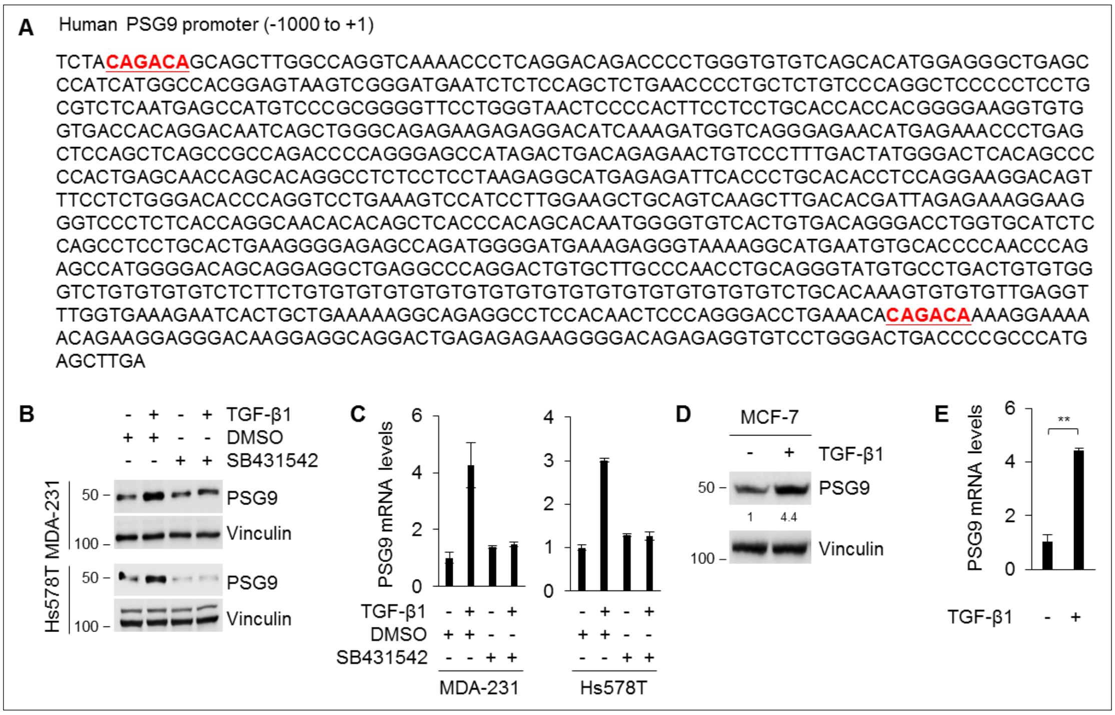

Figure S4. PSG9 is a transcriptional target of TGF-β1

(**A**) The human *PSG9* promoter within 1-kb region upstream from the transcriptional start site contains two canonical Smad-binding elements (SBEs) (CAGACA) [2, 3], located at -996 to -991 and -95 to -90. (**B-C**) MDA-231 and Hs578T cells were cultured in serum-free medium for 24 h and then treated with or without 10 ng/ml of TGF-β1, 10 μM SB431542 alone or in combination for another 24 h. Immunoblotting (B) or qPCR (C) analyses were carried out to detect PSG9 expression levels. (**D-E**) MCF-7 cells were cultured in serum-free medium for 24 h and then treated with or without 10 ng/ml of TGF-β1 for another 24 h. Immunoblotting (B) or qPCR (C) analyses were carried out to detect PSG9 expression levels. In B, the expression levels of PSG9 were normalized to those of Vinculin. $**p<0.01$.

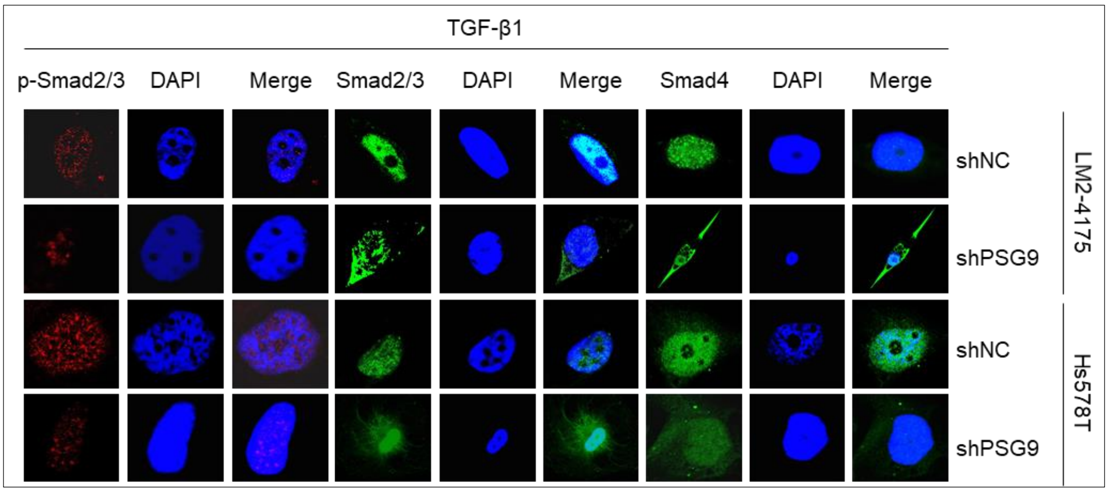

Fig. S5. The effect of PSG9 on the activation of the Smad signaling in response to TGF-β1

LM2-4175 and Hs578T cells stably expressing shNC and shPSG9 were cultured in serum-free media for 24 h, and then treated with or without 10 ng/ml of TGF-β1 for 6 h. Immunofluorescent staining was carried out with the indicated antibodies. Cell nucleus was counterstained with DAPI.

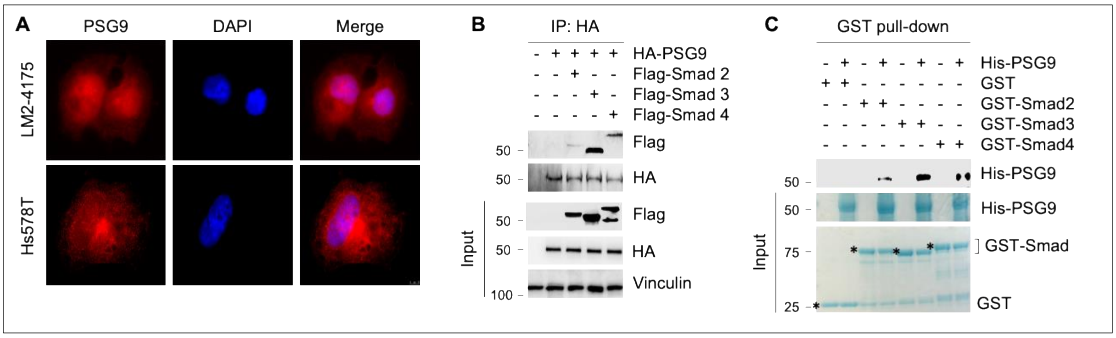

Fig. S6. The subcellular localization of PSG9 and the interaction between PSG9 and Smad2/3/4

(**A**) Immunofluorescence staining of PSG9 in LM2-4175 and Hs578T cells with an anti-PSG9 antibody. Cell nucleus was counterstained with DAPI. (**B**) HEK293T cells were transfected with HA-PSG9 and Flag-Smad2/3/4 alone or in combination. After 48 h of transfection, IP and immunoblotting analysis were performed with the indicated antibodies. (**C**) Reciprocal GST pull-down assays were performed using GST-Smad2/3/4 and His-PSG9. GST was used as a negative control. Immunoblotting analysis was conducted with an anti-PSG9 antibody. GST, GST-Smad2/3/4, and His-PSG9 proteins were visualized by Coomassie blue staining.

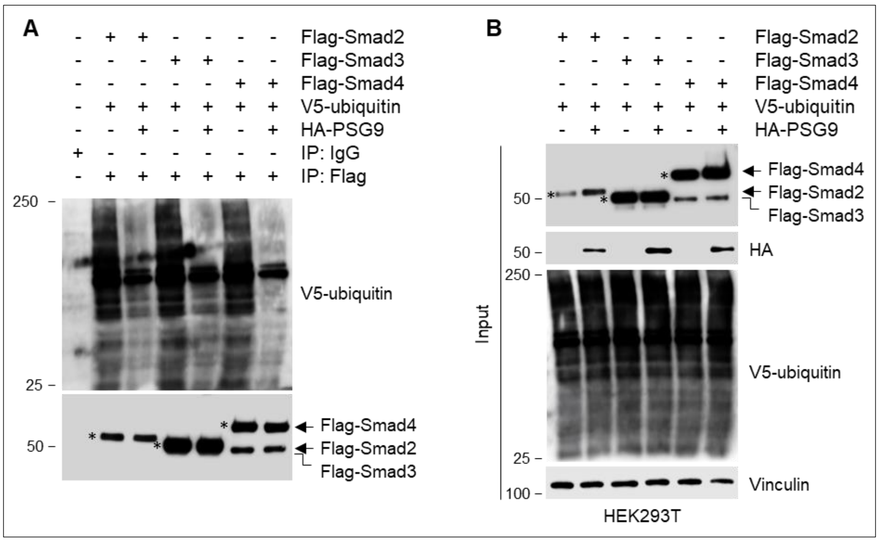

Figure S7. The effect of PSG9 on the ubiquitination of Smad2/3/4

(**A and B**) HEK293T cells were transfected with the indicated expression vectors. After 48 h of transfection, cells were treated with 10 μM MG-132 for 6 h. Lysates were subjected to IP assays, followed by immunoblotting analysis with the indicated antibodies.

Supplementary Tables

Table S1. Primers used for molecular cloning of expression vectors

	Primers	Sequences
HA-PSG9	Forward	CGGAATTCATGGGGCCCCTCCCAG
	Reverse	TCGAGTCGACTCATGACTGAGACTCTGTCAGGTCT
Flag-Smad2	Forward	GCTCTAGAATGGATTACAAGGATGACGACGATAAGATGTCGTCCATCTTGCCATTC
	Reverse	TCGAGTCGACTTATGACATGCTTGAGCAACGC
Flag-Smad3	Forward	GCTCTAGAATGGATTACAAGGATGACGACGATAAGATGTCGTCCATCCTGCCTTT
	Reverse	TCGAGTCGACCTAAGACACACTGGAACAGCGG
Flag-Smad4	Forward	GCTCTAGAATGGATTACAAGGATGACGACGATAAGATGGACAATATGTCTATTACGAATACACC
	Reverse	TCGAGTCGACTCAGTCTAAAGGTTGTGGGTCTG
pGL3-PSG9	Forward	GGGCGGTGTTATTGCTCTTT
	Reverse	GGACTGATCTTGAACTCCTGATCTC
pGL3-PSG9 mut1	Forward	TCTATCGATAGGTACCTCTAAAAAAAGCAGCTTGGCC
	Reverse	TTTGACCTGGCCAAGCTGCTTTTTTTAGAGGTACCTA
pGL3-PSG9 mut2	Forward	ACTCCCAGGGACCTGAAACAAAAAAAAAAGGAAAAAC
	Reverse	CTCCTTCTGTTTTTCCTTTTTTTTTTGTTTCAGGTCC
pGEX6P-1-Smad2	Forward	GGAAGTTCTGTTCCAGGGGCCCCTGGGATCCATGTCGTCCATCTTGCCATTCACGCC
	Reverse	TCGTCAGTCAGTCACGATGCGGCCGCTCGAGTGACATGCTTGAGCAACGCACTGAA
pGEX6P-1-Smad3	Forward	GGAAGTTCTGTTCCAGGGGCCCCTGGGATCCATGTCGTCCATCCTGCCTTTCACTCC
	Reverse	TCGTCAGTCAGTCACGATGCGGCCGCTCGAGAGACACACTGGAACAGCGGATGCT
pGEX6P-1-Smad4	Forward	GGAAGTTCTGTTCCAGGGGCCCCTGGGATCCATGGACAATATGTCTATTACGAATAC

	Reverse	TCGTCAGTCAGTCACGATGCGGCCGCTCGAGGTCTAAAGGTTGTGGGTCTGCAATCG
PET28a(+)-PSG9	Forward	GTTTAACTTTAAGAAGGAGATATACCATGGGCATGGGGCCCCTCCCAGCCCCTTCCTGC
	Reverse	GTTAGCAGCCGGATCTCAGTGGTGGTGGTGGTGGTGCTCGAGTGACTGAGACTCTGTCAGGT

Table S2. siRNA target sequences

		Sequences
siTGFBR1#1	Forward	CACAACCGCACUGUCAUUCTT
	Reverse	GAAUGACAGUGCGGUUGUGGC
siTGFBR1#2	Forward	UUGUUCAGAGAACAAUUGCTT
	Reverse	GCAAUUGUUCUCUGAACAAGC
siTGFBR2#1	Forward	AAGACGCGGAAGCUCAUGGTT
	Reverse	CCAUGAGCUUCCGCGUCUUGC
siTGFBR2#2	Forward	GAGCACUGUGCCAUCAUCCTT
	Reverse	GGAUGAUGGCACAGUGCUCGC
siSMAD2#1	Forward	CCCUGCAACAGUGUGUAAATT
	Reverse	UUUACACACUGUUGCAGGGTT
siSMAD2#2	Forward	GCAGAACUAUCUCCUACUATT
	Reverse	UAGUAGGAGAUAGUUCUGCTT
siSMAD3#1	Forward	GAGUUCGCCUUCAAUAUGATT
	Reverse	UCAUAUUGAAGGCGAACUCTT
siSMAD3#2	Forward	CCGCAUGAGCUUCGUCAAATT
	Reverse	UUUGACGAAGCUCAUGCGGTT
siSMAD4#1	Forward	GCCAUCGUUGUCCACUGAATT
	Reverse	UUCAGUGGACAACGAUGGCTT
siSMAD4#2	Forward	GCCAGCUACUUACCAUCAUTT
	Reverse	AUGAUGGUAAGUAGCUGGCTT

Table S3. Antibodies used in this study

Antibodies	Vendors	Cat#	Hosts	Working concentration
TGFBR1	Abcam	ab31013	Rabbit	1:1000 (WB)
TGFBR2	Abcam	ab186838	Rabbit	1:1000 (WB)
HA	CST	3274S	Rabbit	1:3000 (WB)
Smad2	CST	5339S	Rabbit	1:1000 (WB)
Smad3	CST	9523S	Rabbit	1:1000 (WB)
Smad2	Abcam	ab40855	Rabbit	1:200 (IF)
Smad3	Abcam	ab40854	Rabbit	1:200 (IF)
Smad4	CST	38454S	Rabbit	1:1000 (WB)
p-Smad2	Abcam	ab188334	Rabbit	1:1000 (WB)
p-Smad3	Abcam	ab52903	Rabbit	1:1000 (WB)
E-cadherin	CST	3195s	Rabbit	1:200 (IF)
N-cadherin	CST	13116S	Rabbit	1:200 (IF)
MTA1	CST	5646S	Rabbit	1:1000 (WB)
V5	CST	13202S	Rabbit	1:1000 (WB)
PSG9	Novus	NBP2-19980	Rabbit	1:150 (IHC)
PSG9	Sigma	SAB2701443	Rabbit	1:1000 (WB)<br>1:100 (IF)
Vinculin	Sigma	V9131	Mouse	1:3000 (WB)
Flag	Sigma	F1804-1MG	Mouse	1:3000 (WB)
His	CST	12698T	Rabbit	1:1000 (WB)
GST	GNI	GNI4110-GT	Mouse	1:1000 (WB)

Note: IF, immunofluorescence; IHC, immunohistochemistry; WB, Western blotting

Table S4. Primers for qPCR analysis

	Primers	Sequences
PSG9	Forward	TTGGCAGTAGGGTTTTATGTGG
	Reverse	TGTAGAGACAAATTGGGAGGGT
Snail	Forward	CTTCCAGCAGCCCTACGA
	Reverse	AGCCTTTCCCACTGTCCTC
Slug	Forward	AGACCCCCATGCCATTGAAG
	Reverse	CTTCTCCCCCGTGTGAGTTC
ZEB1	Forward	AGAATTCACAGTGGAGAGAAGCC
	Reverse	CGTTTCTTGCAGTTTGGGCATT
ANGPTL4	Forward	GTCCACCGACCTCCCGTTA
	Reverse	CCTCATGGTCTAGGTGCTTGT
CXCR4	Forward	ACTACACCGAGGAAATGGGCT
	Reverse	CCCACAATGCCAGTTAAGAAGA
IL-11	Forward	CGAGCGGACCTACTGTCCTA
	Reverse	GCCCAGTCAAGTGTCAGGTG
GAPDH	Forward	CTGGGCTACACTGAGCACC
	Reverse	AAGTGGTCGTTGAGGGCAATG

Table S5. Primers for ChIP-qPCR analysis

	Primers	Sequences
PSG9 promoter region1	Forward	TCTACAGACAGCAGCTTGG
	Reverse	GGAGAGATTCATCCCGACTTAC
PSG9 promoter region2	Forward	GAAGAGGCTCAGCACAGAAG
	Reverse	TGCTGCCTGTGTGTTCTC

Table S6. Characterization of clinicopathological features of 161 patients with primary breast cancer (IHC staining of PSG9)

	PSG9 expression		p value
	Low (n=83)	High (n=78)	
Age			0.220
≤50	29	44	
>50	34	34	
Menopausal status			0.948
Premenopausal	36	45	
Postmenopausal	27	33	
Lymph node status			0.023
Negative	38	32	
Positive	25	46	
Grade			0.887
I	1	1	
II	30	38	
III	17	24	
Unknown	15	15	
ER status			0.086
Negative	28	46	
Positive	35	32	
PR status			0.066
Negative	29	48	
Positive	34	30	
HER-2/neu status			0.036
Negative	24	43	
Positive	39	34	

Table S7. Characterization of clinicopathological features of 161 patients with primary breast cancer (detection of plasma PSG9 levels by ELASA)

	PSG9 expression		*p* value
	Low (n=84)	High (n=77)	
Age			
≤50	41	36	0.794
>50	43	41	
Menopausal status			
Premenopausal	50	35	0.500
Postmenopausal	49	42	
Lymph node status			
Negative	58	45	0.0004
Positive	26	32	
Grade			
I	2	2	
II	34	26	0.814
III	28	31	
Unknown	20	18	
ER status			
Negative	26	28	0.468
Positive	58	49	
PR status			
Negative	35	41	0.142
Positive	49	36	
HER2/neu status			
Negative	52	46	0.798
Positive	27	28	
Unknown	5	3	

References

1. Finak G, Bertos N, Pepin F, et al. Stromal gene expression predicts clinical outcome in breast cancer. *Nat Med*. 2008;14(5):518-27. doi:10.1038/nm1764
2. Jonk LJ, Itoh S, Heldin CH, ten Dijke P, Kruijer W. Identification and functional characterization of a Smad binding element (SBE) in the JunB promoter that acts as a transforming growth factor-beta, activin, and bone morphogenetic protein-inducible enhancer. *J Biol Chem*. 1998;273(33):21145-52. doi:10.1074/jbc.273.33.21145
3. Chen SJ, Yuan W, Lo S, Trojanowska M, Varga J. Interaction of smad3 with a proximal smad-binding element of the human alpha2(I) procollagen gene promoter required for transcriptional activation by TGF-beta. *J Biol Chem*. 2000;183(3):381-92. doi:10.1002/(SICI)1097-4652(200006)183:3<381::AID-JCP11>3.0.CO;2-O
